# Green label marinades: A solution to *salmonella* and *campylobacter* in chicken products?

**DOI:** 10.1016/j.heliyon.2023.e17655

**Published:** 2023-07-04

**Authors:** Maitiú Marmion, Arturo B. Soro, Paul Whyte, Amalia G.M. Scannell

**Affiliations:** a UCD School of Agriculture and Food Science, Ireland; b UCD Institute of Food and Health, Ireland; c UCD Centre for Food Safety, Ireland; d UCD School of Veterinary Medicine, University College Dublin, Belfield, Dublin 4 D04 V1W8, Ireland; e Teagasc Research Centre, Ashtown, Dublin 15, Ireland

**Keywords:** Biopreservation, Green label antimicrobials, Food safety, *Salmonella*, *Campylobacter*, Microbial spoilage

## Abstract

**Introduction:**

The presence of meat-borne pathogens entering the home remains a concern for consumers, despite advances made in improving antimicrobial interventions and systems within the processing line. Naturally antibacterial food ingredients including citrus juice and essential oils have been proven to inhibit the proliferation of microbial growth with varying success.

**Aims:**

This study aims to investigate the antimicrobial and sensory effects of mixtures of essential oils, fruit juices and herbs at established Minimum Inhibitory Concentrations (MICs) for their biopreservative effect on general microbiota of chicken and against chicken challenged with selected pathogenic/surrogate microorganisms.

**Materials and methods:**

Three marinade compositions were designed for use on chicken meat; lemon juice, thyme oil and black pepper (M1), lime juice, lemongrass oil and chilli paste (M2), and olive oil, oregano oil, basil oil and garlic paste (M3). These marinades were assessed for antibacterial effects against *Salmonella enterica, Campylobacter jejuni* and *Listeria innocua* on marinaded chicken drumsticks stored in aerobic conditions at 4 °C. Consumer tasting sessions were also conducted with a small focus group using selected final marinades.

**Results:**

M1 and M2 were effective at significantly reducing initial pathogen carriage from 6 Log CFU/g to 2 Log CFU/g on refrigerated chicken meat as well as increasing the shelf-life of the product during cold-storage from 2 days to 7 days. However, consumer studies indicate that the flavours these marinades impart to treated products can be strong.

**Conclusion:**

These findings indicate that these designed marinades have shown excellent potential to improve food safety as well as shelf-life for the consumer, particularly in settings where food safety is often compromised such as barbecuing or in care settings. However, further recipe optimisation is required to make these marinades acceptable to consumers.

## 1 Introduction

Microbial survival and proliferation on meat products is associated with both product spoilage due to *Pseudomonas* spp., Lactic Acid Bacteria (LAB), and *Brochothrix thermosphacta*, and human disease related to pathogens including *Salmonella enterica* and *Campylobacter* spp [[1], [2], [3]]. Meat processors employ a series of ‘hurdles’ such as refrigeration, modified atmosphere packaging, (MAP), washing and scalding to control the product microbiome, and improve product safety, quality and longevity [[4], [5], [6], [7]]. There has been interest in recent years in the development of novel active interventions also, including chemical washes, ultraviolet light, high pressure processing, cold plasma, sonication, and irradiation which have been implemented in some regions [[8], [9], [10]]. However, consumers tend to be reluctant to adopt many of these approaches which limits their usage. This is particularly evident with chemical interventions, with less than 20% of U.K. consumers willing to accept use of chemical decontaminants to reduce *Campylobacter* contamination on broiler meat [11]. There are further legislative hurdles, including European Union prohibition of the use of chemical compounds on fresh meat above concentrations permitted in drinking water [12]. Nonetheless, there remains strong consumer demand for acceptable, safe, natural and effective antimicrobial interventions [11,13,14]. Green consumerism and favouring reduced ‘artificial’ chemicals during production in favour of natural additives exerts a strong influence over product preference [13,15]. This favours the use of Generally Recognised as Safe (GRAS) additives in food production if additives are required. These additives include essential oils, some organic acids, plant extracts and natural compounds like Nisin and chitin. These additives can be added to extend the lifespan of products through prevention of microbial and chemical spoilage processes, or as anti-pathogenic interventions [[16], [17], [18]].

Essential oils (EOs) are produced by plants with the purpose of countering abiotic and biotic challenges, including oxidative stress, heat stress, microbial pathogens, and herbivore and insect activity [19,20]. EOs are expressed in many plant tissues and structures, and tend to have high concentrations of polyphenols, terpinenes, phenylpropanoids and nitrogenous/sulphurous compounds which also produce distinctive tastes and aromas [[19], [20], [21], [22]]. EOs can provide several advantages to the food industry when employed to increase product safety and longevity by acting as antioxidant and antimicrobial compounds. EOs contain unstable double bonds, which allow EOs to act as radical chain quenchers. This reduces the impact of oxidative stress on product quality and longevity [19,23]. This is particularly important with meat products.

EO antimicrobial activity is contingent upon active compounds within the oil, supporting compounds, and the microbial species in question. EO compounds such as thymol, eugenol and carvacrol contribute to the bactericidal effects of essential oils, and are most effective when combined in whole-oil emulsions (Supplementary Table 1) [20]. These compounds can cause phospholipid membrane disruption, leading to cell content leakage, abolishing the proton motive force, and reducing intracellular ATP and DNA replication through effects on DNA gyrase. This affects ATP-dependent cell processes including protein and enzyme synthesis [18,22,24]. This disruption also offers the potential to circumvent efflux-mediated antimicrobial resistance in bacteria [25]. EOs which are high in carvacrol and thymol including thyme and oregano oil have been broadly studied for their potent effects on bacterial growth [14,18,26,27]. However, this can affect Gram-negative and Gram-positive species differently due to the presence of lipopolysaccharide in Gram-negative cell walls which increases tolerance to EOs [28].

Organic acids including citric acid and lactic acid are natural products which can be utilised for the control of bacteria. Used directly on food products, within dips, or as sanitisers, organic acids act to cause cell cytosol acidification and membrane modification through ion incorporation, while organic acid anions also accumulate to toxic levels in bacterial cytosol [18]. Many fruit juices contain citric acid, which accumulates in fruit flesh as citrate as part of the tricarboxylic acid cycle. This is in highest concentration early in the fruit formation process. It is thought that organic acids such as malate and citrate act as carbon storage intermediary compounds during fruit formation, while coordinate fruit maturation as the concentration of organic acid intermediates falls with fruit ripening [29,30]. Some fruits retain a higher concentration of organic acid intermediates when ripe, such as in lemons and limes, where citrate can compose 8% of the dry weight of the fruit, and about 5% of the volume of juice [29,[31], [32], [33]]. As such, the acidity of these juices can be within a pH range of 2–3 which makes them suitable for use in controlling bacterial growth.

Consumer demand for minimally treated foodstuffs has led to a need for the optimisation of available natural antimicrobial compounds. Regulations limiting the use of chemical additives to chicken meat have reduced the suitability of organic acid and other additive use [34]. As such, it has become pertinent to develop antimicrobial approaches based on natural compounds which can be employed without compromising consumer demands or product safety standards.

The aims of this study were to (i) explore the impact of green label antimicrobial interventions on the shelf life of value-added poultry products, (ii) to maximise the impact of these interventions on poultry pathogens, thereby improving consumer safety, and (iii) to determine the consumer acceptability of the marinades on cooked chicken drumsticks.

## 2 Materials and methods

**Fig. 1:**
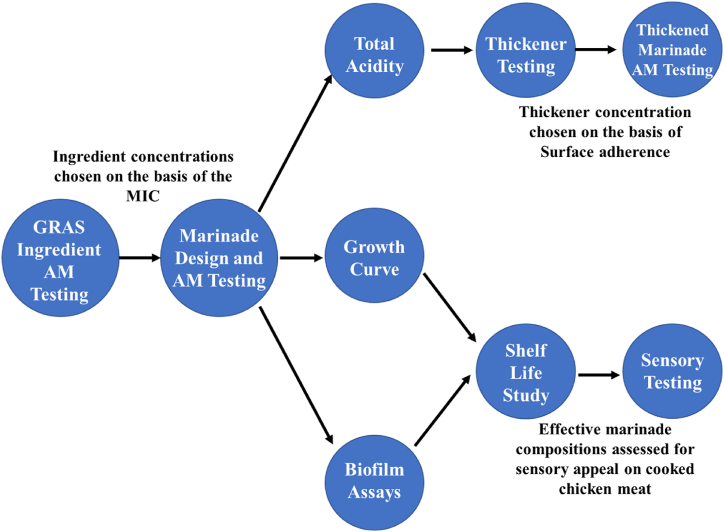
*Overall experimental design.* *GRAS =Generally recognised as safe. AM = Antimicrobial. MIC = Minimum Inhibitory Concentration.

Approaches used in this study were based on previous work by Pedros-Garrido et al. (2020) and Gutierrez et al. 2008 [14,18] (Fig. 1). Agar-well based approaches were used to select promising antimicrobial agents against a variety of production relevant bacterial species. These agents were then employed in growth assays at concentrations at which they had been effective to estimate the effect of these agents on selected bacterial species growth, as well as biofilm formation.

### 2.1 Consumer-led marinade development

A group of 12 consumers who ate chicken regularly, aged between 18 and 50 were recruited at University College Dublin. The group were introduced to the concept of using marinades for adding flavour and Food Safety. In the context of literature-based antimicrobial activity, the panel were introduced to a range of essential oils and acids, and their opinions were sought regarding their linking for the flavours, their purchasing history of seasoned ready-to-cook chicken products on the market, and flavours they would like to see (data not shown). Based on the findings of this focus group and the range of seasoned value-added chicken available in retail settings, and Scientific Literature on the basis of their efficacy against bacterial species, a variety of Generally Recognised As Safe (GRAS) antimicrobial agents were selected with which to develop marinades (Table 1).

**Table 1:** *Antimicrobial agents investigated*.

Antimicrobial Compound	Concentrations employed (% v/v)
Oregano Oil	1.0, 0.8, 0.6, 0.5 0.4, 0.2, 0.1%
Thyme Oil	1.0, 0.8, 0.6, 0.5 0.4, 0.2, 0.1%
Garlic Oil	1.0, 0.8, 0.6, 0.5 0.4, 0.2, 0.1%
East Indian Lemongrass Oil	1.0, 0.8, 0.6, 0.5 0.4, 0.2, 0.1%
Basil Oil	1.0, 0.8, 0.6, 0.5 0.4, 0.2, 0.1%
Citric Acid Lime Juice	10, 5.0, 1.0% 100, 10, 1.0%
Lemon Juice	100, 10, 1.0%

### 2.2 Preparation of antimicrobial agents

Unfiltered essential oils (Sigma-Aldrich (SA), Arklow, Ireland) were diluted to the concentration of interest in sterile 0.5% v/v Tween80 solution (SA, Arklow, Ireland) and stored at room temperature until use. Preliminary data found no significant difference in the antimicrobial effect of unsterilised oils and oils filter sterilised using 22 μm pore filters (P > 0.05; data not printed). Concentrated organic acid stock solutions (10% w/v citric acid; SA, Arklow, Ireland) were prepared, then sterilised by autoclaving (115 °C, 15 min). From this, varied concentrations of acid solutions were prepared in sterile water. Serial dilutions of commercial lemon juice and lime juice were made in sterile water.

### 2.3 Bacterial preparation

Known Gram positive and Gram-negative typed strains were grown overnight under optimal conditions (Supplementary Table 2). Overnight cultures of the bacteria were diluted to an initial optical density (O_D_600) of 0.1 (8–10 Log CFU/ml) and would be used in the following protocols.

### 2.4 Minimum Inhibitory Concentration determination

An adapted protocol was used based on agar-diffusion methods employed for the investigation of antimicrobial effect of natural compounds on bacteria by Balouiri, 2016 [35]. In brief, overnight cultures of the bacteria were diluted to an initial optical density (O_D_600) of 0.1, or 8–10 Log CFU/ml. These cultures were used to inoculate liquid Mueller-Hinton Agar (MHA) cooled to 45 °C (VWR Chemicals, Leuven, Belgium). Once this solidified, the antimicrobial mixture of interest was dotted onto the seeded agar at target concentrations (Table 1). The plates were incubated according to optimal growth conditions for the strains used (Supplementary Table 2), and the zone of inhibition created was measured after an appropriate amount of time. Due to the difference in miscibility of oil compounds and the water-based agar, the zone of clearance size was not deemed to be appropriate in assessing antimicrobial effect, in particular when comparing juice-based and oil-based marinades. The most effective antimicrobial agents/combinations were selected for further use in experiments. This approach was employed for three separate assays.

i) The investigation of essential oil, and juice MICs
ii) The investigation of final marinade effectiveness
iii) The comparison of antibacterial effects of citric acid solutions vs. commercial fruit juices

Final marinade compositions were then designed using focus group-designed flavour combinations before testing final marinade antimicrobial effect. This antimicrobial effect testing was expanded to assess the effect against multiple *Campylobacter jejuni* isolates, *Salmonella enterica* serovar Dublin, and *Salmonella enterica* serovar Enteriditis, as well as the previously stated bacterial species.

### 2.5 Time-kill inhibition curve

An Time-Kill kinetics assay based on the work of Appiah et al., 2017 was employed to investigate the duration of survival of test pathogens in the final marinade solutions [36]. *Salmonella enterica* serovar Typhimurium, *Campylobacter jejuni* or *Listeria innocua* were grown overnight as appropriate (Supplementary Table 2) These bacteria were diluted to 0.1 O_D_600 (8–10 Log CFU/ml), and pelleted at 10,000 x G. The pellet was washed once in sterile Ringer solution, then re-pelleted. This was re-suspended in the proposed antimicrobial marinades (Table 2) and placed in a shaking incubator at 37 °C. Cell enumeration of 1 ml aliquots of the inoculated marinade was carried out on Xylose Lysine Deoxycholate (XLD), modified Charcoal Cefoperazone Deoxycholate Agar (mCCDA) or Ottaviani-Agosti *Listeria* selective agar at the 0, 2, 4, 6, 8 and 24 h timepoints to capture cell survival under the given conditions.

**Table 2:** GRAS-marinade compositions.

Marinade	EO Component	EO Concentration (% v/v)	Non-EO component	Flavour Component
1 (M1)	Thyme Oil	0.50	Lemon Juice	Ground black pepper
2 (M2)	Lemongrass oil	1.00	Lime Juice	Chilli paste
3 (M3)	Basil oil Oregano oil	1.00 0.50	Olive oil	Garlic paste

### 2.6 Biofilm formation

*S. enterica* ser. Typhimurium, *E. coli*, *L. innocua*, *B. subtilis* and *C. jejuni* were grown overnight as appropriate (Supplementary Table 2). The overnight culture was pelleted at 10,000 x G, and the supernatant was discarded. The pellet was washed in sterile 1/4-strength Ringer solution. The pellet was then resuspended in the antimicrobial mixture of interest (Table 2) and used to inoculate a polystyrene 96-well plate. These plates were incubated at 37 °C and 4 °C under aerobic and low oxygen conditions. Following incubation for 24 h, the supernatant was removed and was stained according to Tram, 2020 [37], using a 1% (v/v) solution of crystal violet. After washing twice in sterile water, the stain was removed with a 20% acetone solution in ethanol. The O_D_600 of the plate wells was recorded. The following logical parameter was used when tabulating results; =IFS([OD600] < 0.05,”-“, [OD600] < 0.15,”+”, [OD600] < 0.35,”++”, [OD600] < 0.7, “+++”, [OD600] < 1,”++++”, [OD600] > 1, “+++++”).

### 2.7 In-situ microbial challenge testing on chicken meat

Overnight cultures of *Salmonella enterica* serovar Typhimurium, *Listeria innocua* or *Campylobacter jejuni* were prepared in tryptone soya broth (TSB) or TSB with *Campylobacter* Selective Supplement prior to the challenge test. These overnight cultures were pelleted at 13,000×*g*, and, once the supernatant was discarded, resuspended in sterile water to allow individual poultry drumsticks to be inoculated at a cell density of 10^6^ CFU/g.

Fresh chicken drumsticks were purchased on the date of arrival in store. These drumsticks were weighed prior to treatment. The skin of samples was inoculated with the selected bacterial suspension at cell density of 10^6^ CFU/g and spread thoroughly with a sterile spreader. The inoculated surface was allowed to dry. Selected drumsticks were immersed in one of the three optimised GRAS-marinades (Table 2). These drumsticks were stored at 4 °C in a covered container until the time of microbial sampling. Untreated but inoculated drumsticks were adjacently stored for use as a control.

Chicken meat was sampled on days 0, 2, 4, 7 and 10. On the day of sampling, the treated and untreated chicken were removed from chill storage. The chicken drumsticks were rinsed in 100 ml of ¼ strength Ringer solution and placed on a shaking incubator for 10 min 10-fold dilutions of the rinsate were then prepared in Ringer solution. Dilutions of sample rinsates were then plated into general and selective media (Supplementary Table 3) to enumerate bacteria present on the chicken drumstick. Samples were also assessed for the qualitative presence of test pathogens *Salmonella enterica* and *Campylobacter jejuni* using Rappoport-Vassiliadis and Bolton selective broths respectively according to International Standards Organisation (ISO) processes ISO6579-1 and ISO10272-2.

### 2.8 Total titratable acidity

Final marinade compositions were titrated against the 0.1 M Sodium Hydroxide until the pH of the solution reached 9.2, or the colour change point of the phenolphthalein indicator. Results were recorded and reported in terms of cm^3^ of sodium hydroxide.

### 2.9 Thickening and emulsifying agent selection

A selection of four thickening agents with emulsifying properties were incorporated into marinade compositions (Table 2) at similar concentrations to those employed in commercial products (Supplementary Table 4). These products were analyzed using an Ametek Brookfield DV1 Viscometer (Middleboro, MA, U.S.A) upon product mixing, and after storage at 4 °C for 24 h. Marinade compositions were chosen based on producing a viscosity between 100 and 1000 cP (cp) to ensure adherence of the treatments to chicken surfaces. Due to improved thickening activity of gelatin at a neutral pH, pH adjusted marinades were also made through the addition of sodium bicarbonate to citrus juice-based marinades prior to the addition of thickeners. Functional adherence properties of the thickened marinades were also assessed using a chef test to determine for their ability to adhere to a smooth stainless-steel. Specifically, a metal tablespoon was dipped in the mixture. The spoon was then held up horizontally, with the back of the spoon facing upward. A finger was then traced across the spoon to create a path through the mixture. Marinade formulations with optimal viscosity and that retained the traced path were selected for use in antimicrobial susceptibility and biofilm formation assays against selected pathogens as described above.

### 2.10 Sensory evaluation

Ethical approval was granted by the 10.13039/501100001631University College Dublin Board of Ethics in February 2023 (Ref No: LS-23-01-Marmion-Sca). A panel of consumers (n = 48) was recruited. Retail chicken breast was selected for this study as it allowed sample uniformity compared to drumsticks. This was purchased and marinated for 3 h at 4 °C in M1 or M2. The chicken was cooked to an internal temperature of 72 °C according to American Meat Science Association (AMSA) guidelines and served to the tasting panel [38]. Samples were blind-coded and randomised, then served monadically to the tasting panel. Consumers answered questions about their overall liking of the product appearance, aroma, flavour, and overall liking on a 9 point scale where 1 = extremely dislike, and 9 = like extremely Additional questions probing consumers impression of the recipe optimisation were also included, describing their perception of [1]. attribute intensity using a 1–9 scale were 1 = absent/not at all and 9 = extremely intense; and [2] flavour propriety, using a 5 point, just-about-right (JAR) scales, where 3 was given as the assessor's ideal or ‘just about right score.

After completing one sample, a timer prevented assessors moving to the next sample before a 1-min wait period was completed. During this time assessors were asked to rinse their mouths with water, eat a cream (water) cracker (Jacobs, UK) and rinse the mouth again until the cracker was cleared to avoid carry over of flavours to the next sample [39]. Data was captured electronically on RedJade® Software.

### 2.11 Statistical analysis

IBM SPSS Version 27 software was utilised to perform a series of statistical tests to assess the effectiveness of the marinades designed. The difference in efficacy of citrus juices versus dilute citric acid solutions was established by means of independent sample Student's t-test. Differences between mixture efficacy and biofilm formation was assessed using multivariate analysis of variance (MANOVA) testing. This software was further employed to determine the best Green Dip compositions via mean Minimum Inhibitory Concentrations (MIC) determinations, and to investigate the changes in the chicken meat microflora through ANOVA analyses. Microsoft Excel Version 16 was used to collate and graph initial data as required. Differences in the sensory data were explored using 2-way ANOVA followed by Tukey's T-Test where significant divergences (P < 0.05) were observed.

## 3 Results and discussion

The challenge posed by the poultry meat microbiota necessitates the employment of antibacterial approaches prior to sale and consumption. This study sought to design safe to consume marinades composed of antimicrobial components which would effectively reduce risks posed by bacterial contamination of chicken meat. Essential oils have been a popular intervention proposal, given their effectiveness against bacterial proliferation, their natural status in the minds of many consumers, and their relative cost-effectiveness, while their role as a dominant sensory determinant of many plants and herbs make them suitable for use in flavour additives (Supplementary Table 1).

Bacteria can exist as both planktonic cells and matrix-bound biofilm communities within the natural world including on meat surfaces. Thus, it was important to assess the response of a range of Gram-positive and Gram-Negative species to the proposed antimicrobial ingredients and marinade compositions, especially given the noted differences in growth conditional susceptibility within these disparate modes of living [40]. From these assessments, effective concentrations of essential oils were selected for the development of antibacterial marinades using the determined MIC, and the effect on bacterial carriage on chicken meat inoculated with pathogenic bacterial isolates was determined.

### 3.1 Evaluating the antimicrobial effect of essential oils and citrus juices against Gram-positive and Gram-negative bacterial species

The bacterial microbiota of broiler meat is a diverse entity, composed of Gram-positive and Gram-negative species which were derived from both the broiler microbiome and the processing facility microbiota [41]. As noted, these microbial assemblages play a crucial role in both product safety and quality. Due to this diversity, a representative selection of Gram-positive and Gram-negative species (Supplementary Table 2) were treated with proposed ingredients to assess the effectiveness of these ingredients against bacterial growth.

The most broadly effective essential oils were Thyme oil and Oregano oil, with MICs of 0.1–0.5% (v/v) against Gram-positive organisms, and 0.5–1.0% (v/v) against Gram-negative organisms. With the exceptions of tests against olive and garlic oil, Gram-positive organisms such as *Listeria innocua*, *B. thermosphacta* and *Bacillus subtilis* showed high susceptibility to the EOs employed, particularly thyme and oregano EOs which contain thymol and carvacrol. These oils were effective at MICs as low as 0.1% (v/v) against Gram-positive species. Gram-negative organisms showed more resilience when grown in the presence of these oils, which MIC of 0.5% (v/v) oregano and thyme oil needed to prevent the growth of all tested bacteria (Table 3). As a result, this EO concentration was incorporated into the final marinade composition to address the more resilient Gram-negative species which might be present in food. Other EOs such as lemongrass, garlic oil and basil oil were not as effective against the selected microorganisms, with higher MICs of up to 1.00% needed to prevent the growth of a wider range of bacterial species.

**Table 3:** Minimum inhibitory concentrations of essential oils employed against select bacterial species (% (v/v)).

Bacterial Species	Gram Type	Oregano Oil	Thyme Oil	Lemongrass Oil	Garlic Oil	Basil Oil
*B. subtilis*	G +	0.1	0.1	0.5	1.0	>1.0
*B. thermosphacta*	G +	0.2	0.6	>1.0	>1.0	>1.0
*C. jejuni* Aerobic	G –	0.5	0.5	>1.0	>1.0	>1.0
Microaerobic	G –	0.5	1.0	1.0	>1.0	1.0
*E. coli*	G –	0.1	0.1	1.0	>1.0	>1.0
*L. innocua*	G +	0.5	1.0	>1.0	0.5	>1.0
*P. aeruginosa*	G –	0.5	0.5	>1.0	0.1	1.0
*P. flourescens*	G –	0.5	0.5	0.5	0.5	1.0
*S. enterica* ser. Typhimurium	G –	0.1	0.1	0.5	>1.0	1.0

The MICs found for thyme oil and oregano oil were broadly in line with the findings of other studies in the field, with the clearest effect seen in Gram positive species at concentrations as low as 0.1% (v/v). However, the MIC established in this study was a bit higher than seen in other studies, which was often as low as 0.1% (v/v) against Gram-negative species such as *Salmonella enterica* [18,42,43]. Lemongrass oil showed effect against five of the eight species tested at concentrations as low as 0.5% (v/v), while Basil oil at 1% (v/v) was effective against four species of interest. Garlic oil showed effect against only three species of interest and was not chosen to be incorporated into the end-products This difference in effect may be explained due to the variance in antimicrobial components found in these EOs between sources and crops, with concentrations of thymol, carvacrol, eugenol and similar antimicrobial substances varying by geography, climate, soil and plant variety [26,44]. Due to the strong sensory properties of garlic and basil oil at the indicated MIC for many bacteria, these oils were included at sub-MIC levels to impart taste and aromas to the final compositions of the marinades [23,45].

### 3.2 Comparing the antimicrobial efficacy of fruit juice to citric acid solution

Acidic stress is a common means of controlling bacterial growth that is frequently employed in sanitisers and food preservatives. Furthermore, foods such as Brazilian ceviche employ a mixture of lemon and lime juice to prepare, flavour and sanitise raw fish prior to consumption [[46], [47], [48], [49]]. Lemon and lime juice are naturally acidic substances, containing citric acid at a concentration of around 5% (v/v) with a pH recorded in this experiment of 2.1–2.3 [31,32]. To assess if the low pH of these juices was the principal determinant of the antibacterial effect possible with these juices, the antibacterial growth effect of these juices was compared to the effect of 10, 5 or 1% (v/v) solutions of citric acid. The pH of juices sampled was around 2.1, similar to a 1% citric acid solution. However, this concentration of citric acid did not effectively inhibit a bacterial growth. Undiluted lemon and lime juice were effective against bacterial growth, and showed the same effect as a 5% (v/v) citric acid solution, which had a pH of around 1.9, against *L. innocua* and *E. coli*. This indicates that non-acid juice components such as fruit specific essential oils and natural compounds including d-limonene, β-pinene and citral may improve juice antimicrobial activity, as noted in previous studies [31,32,50]. Certainly, a significant improvement (P < 0.05) in the antibacterial effect was observed in citrus juices when compared to citric acid alone.

### 3.3 Effect of marinade compositions on bacterial growth

**Fig. 2:**
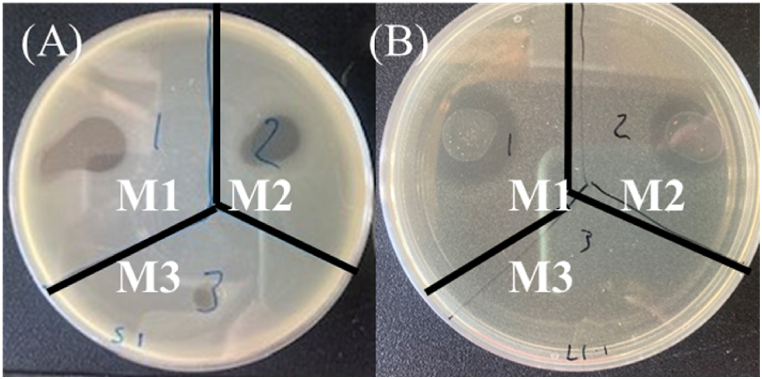
Zones of clearance created by Marinade 1, 2 or 3 in in vitro testing of (A) Salmonella enterica ser. Enterica and (B) Listeria innocua.

Three marinade compositions were formulated based upon EO MICs, as well as the general effectiveness of citrus juices against bacterial growth. Oregano and thyme EOs, as the most effective oils against all challenge species, were chosen for marinade design based on focus group feedback, while lemongrass and basil EOs were chosen because of a combination of focus group recipe suggestions as well as complementation with a strong antimicrobial ingredient in the final marinade (lime juice and oregano EO respectively). Marinades 1 and 2 (M1, M2) showed efficacy against all species of interest under all conditions, while Marinade 3 (M3) only showed some effect against *Pseudomonas aeruginosa* and *S. enterica* ser. Enterica, while remaining ineffective against other species such as *E. coli* and *L. innocua* (Table 4; Fig. 2 (A) and 2 (B)). Further testing was carried out against 8 *Campylobacter jejuni* isolates of clinical and food-processing origin, with similar results in marinade efficacy seen (Supplementary Table 5). M3 further showed some effect against one clinical *Campylobacter jejuni* isolate.

**Table 4:** Antibacterial effect of Final Marinades against selected bacterial species (% (v/v)). ‘+’ indicates formation of a zone of growth inhibition. ‘–‘ indicates no observed antimicrobial effect.

Bacterial Species	Gram Type	Marinade 1	Marinade 2	Marinade 3
*B. subtilis*	G +	+	+	-
*C. jejuni* Aerobic	G –	+	+	-
Microaerobic	G –	+	+	-
*E. coli*	G –	+	+	-
*L. monocytogenes*	G +	+	+	-
*L. innocua*	G +	+	+	-
*P. aeruginosa*	G –	+	+	+
*P. flourescens*	G –	+	+	-
*S. enterica* ser. Dublin	G –	+	+	-
*S. enterica* ser. Enterica	G –	+	+	+
*S. enterica* ser. Typhimurium	G –	+	+	-

### 3.4 Time-kill assay in antimicrobial marinades

**Fig. 3:**
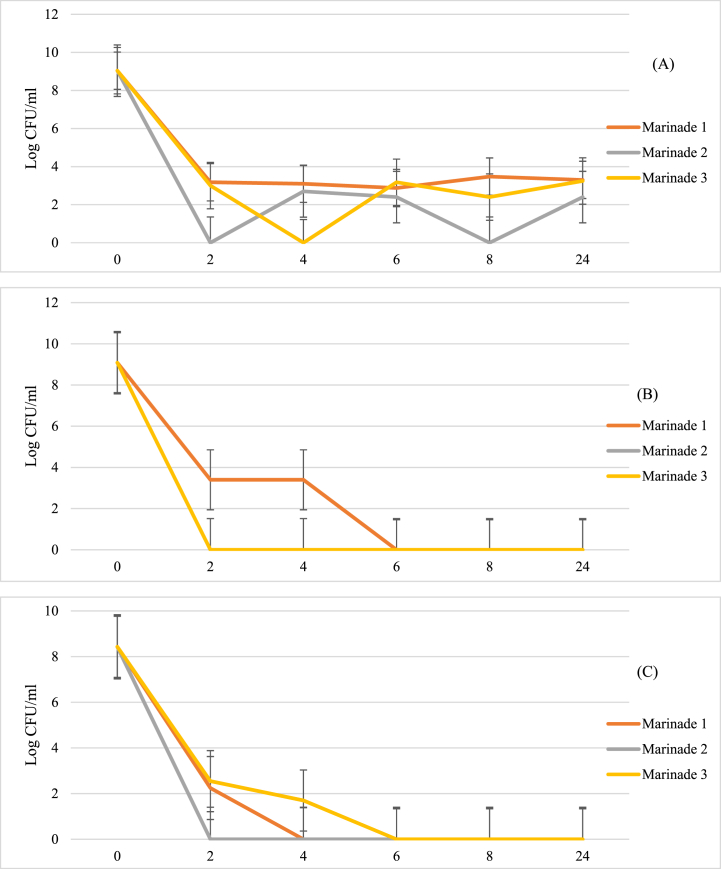
Reductions in growth of (A) Listeria innocua, (B) Salmonella enterica ser. Typhimurium and (C) Campylobacter jejuni suspensions when stored in marinade solutions over a 24-h period. Error bars represent Standard Error.

To determine a more accurate measure of marinade efficacy against selected test strains, in vitro challenge tests, were conducted against *L. innocua*, *S. enterica* ser. Typhimurium and *C. jejuni*. Storage in these marinades led to a significant reduction (P < 0.05) in cell recovery of over 6 log CFU/ml for *L. innocua* and *S. enterica* ser. Typhimurium (Fig. 3A and B), and over 5 log CFU/ml for *C. jejuni* (Fig. 3C). The decline in cell abundance occurred quickly in all species, with a clear reduction seen within 2 h of exposure. M2 showed the fastest and most consistent effect, while slower effect was seen in M1 and M3, depending on the bacterial challenge. Both *Salmonella* and *Campylobacter* were not recoverable within 6 h of exposure to all marinades, but the Gram-positive bacterium *L. innocua* exhibited more tolerance to the marinade challenge. This may possibly be due to its thicker cell wall as a Gram-negative organism, or its conditional tolerance as an environmentally-tolerant microbe [51].

### 3.5 Biofilm analysis

Biofilms are a predominant means of bacterial survival on meat and production surfaces, as well as a key response for conditionally stressed cells. Furthermore, changes in metabolism growth patterns within the biofilm-bound cells are known to reduce the effectiveness of antimicrobial compounds [52]. The results of these assays indicate that biofilm formation is either unaffected or increased when stored in the marinades of interest when compared to control samples for all species (Table 5). Higher biofilm formation was associated with storage at the higher temperature used here for all species under observation. Significantly increased (P < 0.05) biofilm formation was especially seen with storage in M2 at 37 °C, with this increase seen in *Salmonella*, *L. innocua* and *E. coli* under both aerobic and microaerobic conditions. M3 caused significant increases (P < 0.05) in *Campylobacter* biofilm formation under all conditions. M1 caused a significant increase in biofilm formation in *L. innocua* at 37 °C microaerobic conditions alone.

**Table 5:** Biofilm formation in test bacteria when stored in Marinades 1, 2 or 3 under different conditions. * indicates a significant difference (P < 0.05) in biofilm formation after ANOVA testing.

Species	Temp (°C)	Oxygen Availability	H_2_O	MRD	M1	M2	M3
*S. enterica* ser. Typhimurium	4 °C	Aerobic	+	+	++	+++*	++
		Microaerobic	+	+	+	++*	++
	37 °C	Aerobic	+	+	+++	++++	+++
		Microaerobic	+	+	+++*	+++*	++
*L. innocua*	4 °C	Aerobic	+	++	++	+++	+++
		Microaerobic	+	++	+	++	++*
	37 °C	Aerobic	+	++	++	+++*	+++
		Microaerobic	++	++	++	+++*	+++
*E. coli*	4 °C	Aerobic	++	++	++	+++	++
		Microaerobic	+	+	++	++	++
	37 °C	Aerobic	+	+	+++	+++*	+++
		Microaerobic	++	++	+++	+++*	+++*
*B. subtilis*	4 °C	Aerobic	+	+	++	+++*	++*
		Microaerobic	+	+	+++	+++	+++
	37 °C	Aerobic	++	++	++	++	++
		Microaerobic	+	+	++	+++	+++
*C. jejuni*	4 °C	Aerobic	+	+	++	++	++*
		Microaerobic	++	+	++	++	+++*
	37 °C	Aerobic	+	+	+	++*	+++*
		Microaerobic	++	++	++	++	+++*

**‘+’ and ‘-‘ indicate the growth levels as determined by O**_**D**_**600 spectraphotometric values where “***--"* = < 0.05. “+” = < 0.15. “++” = < 0.35. “+++” = < 0.7. “++++” = < 1. “+++++” = > 1.

### 3.6 Bacterial challenge test in situ on chicken meat

**Fig. 4:**
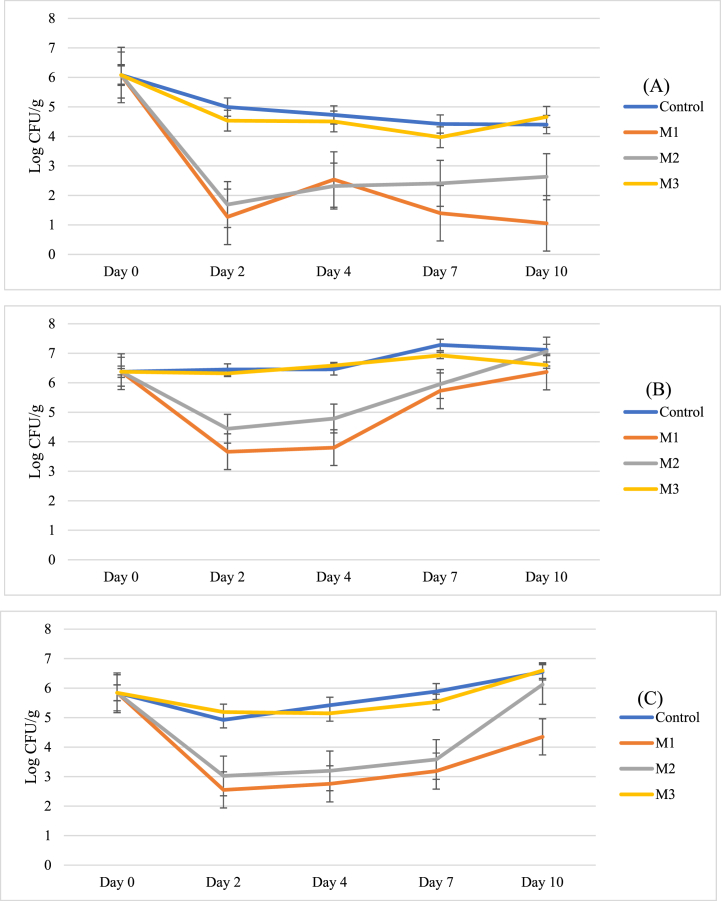
Carriage of artificially inoculated (A) Campylobacter jejuni, (B) Salmonella enterica ser. Typhimurium and (C) Listeria innocua declined significantly (P < 0.01) from starting concentrations when treated with Marinade 1 and Marinade 2. Error bars represent Standard Error.

A significant reduction in the prevalence of *C. jejuni*, *Salmonella* Typhimurium and L. *innocua* was observed throughout the study in meat treated with M1 and M2, with a 5.8 log CFU/g reduction in *Salmonella*, 6.1 log CFU/g reduction in *Campylobacter* and a 6.4 log CFU/g reduction in *L. innocua* after 2 days. No significant reduction was achieved in meat treated with M3 when compared to the untreated control (Fig. 4). *C. jejuni* showed consistent reductions in recoverable bacteria for the period of this study in meat treated with citrus-juice based marinades, with recoverable levels of *Campylobacter* never exceeding 1000 CFU/g (Fig. 4A). However, this trend was not observed in the case of *Salmonella enterica* serovar Typhimurium*.* Though an initial steep decline in the concentration of *Salmonella* was observed, the levels of recoverable *Salmonella* bacteria in treated chicken slowly recovered. This recovery was less pronounced in meat treated with a mixture of lemon juice and thyme oil (M1) (Fig. 4B). *L. innocua* abundance declined significantly within 48 h of inoculation on chicken treated with M1 and M2, but recovery of *L. innocua* increased after Day 7, with bacteria on meat stored in M2 showing quicker recovery than M1 (Fig. 4C).

### 3.7 Shelf-life assessment of treated meat

The microbial limit for aerobic bacteria on mechanically separated or poultry meat is 6.5–7 Log CFU/g, after which it becomes unfit for human consumption [53]. At this point, meat often can be unpalatable and spoilage-related aromas/compounds are notable, both of which relate to microbial overgrowth on the meat matrix [1]. This overgrowth is characterised by fast growth as the bacterial population enters the ‘log’ phase of growth, having adapted to the environmental conditions. It thus becomes important to extend the lag, or adaptive, phase of growth, in order to extend product shelf-life [54].

**Fig. 5:**
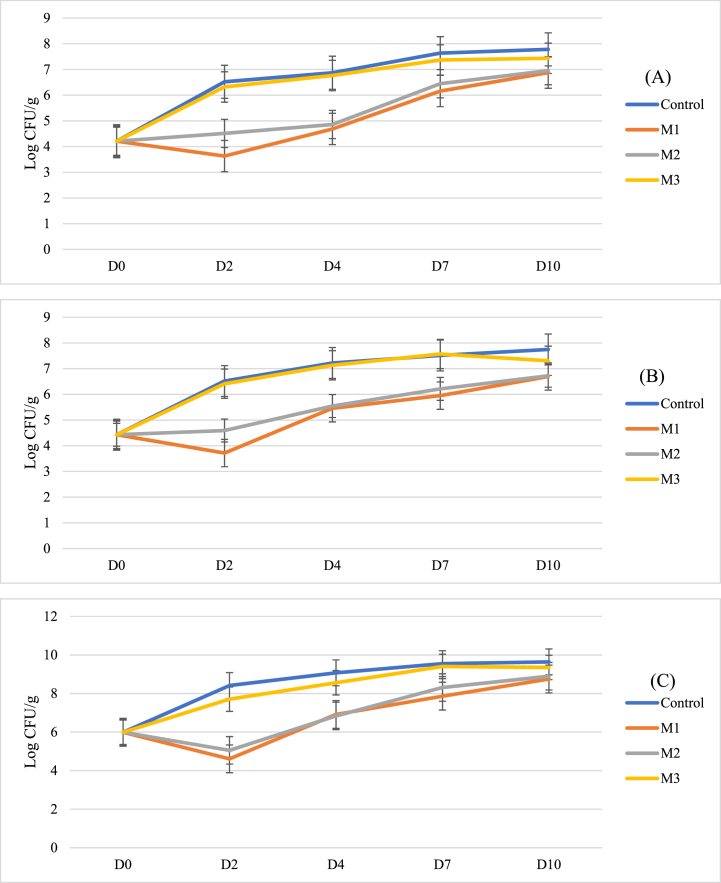
Carriage of (A) Mesophiles, (B) Lactic Acid Bacteria and (C) Psychrophiles on treated and control meat products. Error bars represent standard error.

Chicken meat stored under refrigerated conditions in the designed marinades was thus assessed for the growth of spoilage-related bacterial groups including mesophiles, psychrophiles and lactic acid bacteria (LAB). Citrus juice-based marinades (M1 and M2) were effective in extending the lag phase of microbiota growth, reducing the proliferation of bacteria and resulting in an increase in product shelf life as bacterial numbers remained below 10^7^ CFU/g, from Day 4 to Day 10 for mesophile count (Fig. 5A), and from Day 2 to Day 4 by psychrophile count (Fig. 5C). Both showed significantly less growth (P > 0.01) in meat treated with antibacterial marinades M1 and M2. LAB, an important cause of meat spoilage, remained at below 10^7^ CFU/g for greater than 10 days in meat marinaded in the lemon juice-based marinades (Fig. 5B). However, the difference at this point between treated and untreated chicken microbiota was not significant, even in the effective marinade compositions M1 and M2. M3, however, showed no significant difference in effect on the recoverable bacteria from chicken meat over the shelf-life of the product, with the product spoiling at a similar point in time to the untreated control product, on Day 4 by mesophile counts, and Day 2 by psychrophile counts. No significant difference was observed between the olive-oil based marinade and the control group for either microbial grouping.

### 3.8 Effect of thickening agents on the efficacy of marinades

Common emulsifying and thickening agents were investigated and incorporated into the final marinades in order to address concerns regarding marinade adherence to product surfaces, as well as concerns regarding the miscibility of polar and non-polar elements of the final marinade compositions. It was thought that the differing densities of the olive oil or juice base and EO additives may have affected the miscibility of the EO compounds in these bases, reducing their effect on bacteria present. To address this issue, both emulsifying and thickening agents were investigated and incorporated into the final marinades, and the changes in marinade thickness and mixing was evaluated. Furthermore, the incorporation of these components can aid marinade adherence to surfaces, which is of practicable benefit to the marinade in a production context.

Four commonly-employed thickeners/emulsifiers were investigated for their effect on the viscosity and miscibility of the marinade compositions once MICs and final compositions were determined. To accommodate the effect of refrigeration on solution thickness, these values were measured immediately and after storage for 24 h at 4 °C. Viscosity values of 100–1000 were deemed preferable for the binding of marinades to meat surfaces. Thickening with gelatin and carrageenan are also noted to show functional improvements at a neutral pH when compared to acidic conditions, which required investigation incorporation of sodium bicarbonate to provide a pH neutral environment for the full thickening effect of these compounds when incorporated into the marinade products.

**Fig. 6:**
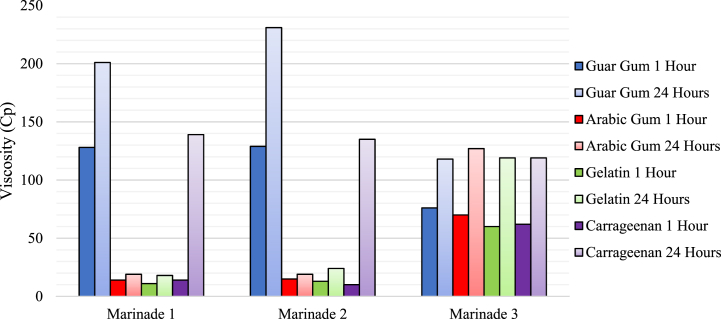
*Viscosity of marinades thickened with Guar gum (0.5% (w/v)), Arabic gum (5% (w/v)), Gelatin (1.5% (w/v)) and Carrageenan (1% (w/v)) during storage at 4 °C*.

Marinades thickened with guar gum at 0.5% (v/v) showed the best immediate performance in an adhesion test on stainless steel, while immediate rheometer measurements and those recorded after storage at 4 C for 24 h showed the desired effect for binding in marinades thickened with 0.5% (w/v) guar gum. (Fig. 6). The Total Titratable Acidity (TTA) of M1 and M2 were 221.37 ± 1.48 cm^3^ and 202.3 ± 2.26 cm^3^ respectively, reinforcing that these citrus juice-based marinades were high acidity foods. Concerns thus arose that this may impede the thickening effect of marinades made with gelatin and carrageenan (Supplementary Table 1), pH neutral marinades were made using sodium bicarbonate. Due to the shared physicochemical properties of lemon and lime juice, a comparison of pH adjusted M2 with unadjusted M2 was made with no inferiority in viscosity seen when assessed with a rheometer. The olive oil based M3 reported viscosity values within the desired range after 24 h of storage, and thus thickening was deemed unnecessary.

Guar gum was selected as the appropriate thickening agent for M1 and M2, with M3 showing appropriate viscosity of between 100 and 1000 cp for binding to meat surfaces without thickening. Thickened M1 and M2 were again assessed for their ability to restrict bacterial growth when compared to unthickened controls after 24 h of incubation. No reduction in antibacterial activity seen when mixtures were thickened with Guar gum and gelatin alone (Table 6). However, thickening with pH-adjusted gelatin-thickened marinades did not show consistent antibacterial results. These results indicate the importance of a low pH in reducing the growth of bacteria in these marinades, with the essential oil component only playing a supplementary role. Thickened marinades also did not significantly affect marinade efficacy (P > 0.05) against biofilm formation in polystyrene surfaces at 4 or 37 °C (Supplementary Fig. 1).

**Table 6:** Antibacterial effect of thickened marinades against selected bacteria. ‘+’ indicates antimicrobial effect. ‘–‘ indicates no observed antimicrobial effect.

Species	Marinade 1						Marinade 2					
	C	O	G	GO	Gl	GlA	C	O	G	GO	Gl	GlA
*Bacillus subtilis* DSM10	+ + +	+ + +	+ + +	+ + -	+ + +	+ + +	+ + +	+ + +	+ + -	+ + +	+ + +	+ + +
*Campylobacter jejuni* NCTC11168	+ + +	+ + -	+ + +	- - +	- - +	- - +	+ + +	- - +	+ + +	+ + -	+ + +	- - -
*Escherichia coli* ATCC8739	+ + +	+ + +	+ + +	- - -	+ + +	+ + +	+ + +	- - +	+ + -	- - +	+ + +	- - -
*Listeria innocua* DSM20649	+ + +	+ + -	+ + -	+ - -	+ + -	- - +	+ + +	- - +	+ + -	+ + -	+ + +	- - -
*Salmonella enterica* ser. Typhimurium ATCC14028	+ + +	+ + +	+ + +	- - -	+ + +	+ + +	+ + +	+ + +	+ + -	- - +	+ + +	+ + +

*C = Control; no thickener. O = 50% Olive Oil. G = Guar Gum. GO = Guar Gum thickened, 50% Olive Oil. Gl = Gelatin. GlA = pH Adjusted (neutral) Gelatin.

While acidic marinades maintained their effect in stopping bacterial growth, though to a lesser extent than the untreated control, marinade neutralisation reduced the efficacy of M2, which utilised the less effective lemongrass EO alongside the citric acid component. This demonstrates that the low pH of this lime juice-based mixture was the principal determinant of its antimicrobial effect. Marinade 1, however, retained some antimicrobial effect, indicating that the EO component thyme oil made some contribution to the overall efficacy of the mixture. Taking these results together, the combined pH and membrane stresses that the marinade components apply to the cell, in concert with chilled storage stress, act to improve the efficacy of the marinades. This employment of multiple hurdles allows increased effect against the bacterial risk at hand.

Biofilm formation by bacteria stored in thickened marinades was also evaluated at 4 °C and 37 °C. No significant difference was seen in biofilm formation when stored in thickened marinades under chilled conditions (4 °C) for all species studied (Table 7). However, storage at 37 °C led to increased biofilm formation for all species studied. Further, *L. innocua* showed significantly increased biofilm formation at 37 °C when stored in thickened M1, and *B. subtilis* showed significantly increased biofilm formation in thickened M2 (P < 0.05).

**Table 7:** Biofilm formation of test bacteria on polystyrene in thickened Marinades 1 and 2 when stored under different conditions. * indicates a significant difference (P < 0.05) in biofilm formation after ANOVA testing.

Species	Temp. (°C)	M1	M1T	M2	M2T
*S. enterica* ser. Typhimurium	4 °C	++	++	+++	+++
	37 °C	+++	+++	++++	+++++
*L. innocua*	4 °C	++	++	+++	++
	37 °C	++	++++ *	+++	+++
*E. coli*	4 °C	++	++	+++	++
	37 °C	+++	+++	+++	+++
*B. subtilis*	4 °C	++	++	+++	++
	37 °C	++	++	+++	+++++ *

‘+’ and ‘-‘ indicate the growth levels as determined by O_D_600 spectraphotometric values where.

“*—"* = < 0.05. “+” = < 0.15. “++” = < 0.35. “+++” = < 0.7. “++++” = < 1. “+++++” = > 1.

Olive oil was also investigated as a thickening additive, given both the good surface adhesion results seen with the olive-oil based marinade M3, as well as the aforementioned need for the dilution of the strong citrus juice flavours noted by the tasting panel which might be achieved with the dilution step. However, marinades thickened with olive oil showed some reduction in efficacy against some bacterial species under investigation such as *Campylobacter*, which tends be present at higher abundances on chicken meat than *Salmonella* (Marmion et al., 2023; unpublished at time of writing). This may be of concern for those with an interest of controlling pathogen contamination from the point of manufacture, though less so for those employing these marinades on products with a lower level of contamination.

### 3.9 Sensory assessment of marinated chicken products

Sensory bench testing in the form of an initial pilot study of 10 sensory panellists determined that marination overnight (12 h) imparted too strong a flavour to the chicken product, and the marination protocol was revised to 3 h (Data not shown). Using the revised protocol, marinated chicken was assessed by a naïve panel of 47 consumers addressing questions on product attributes associated with flavour, aroma, appearance, colour, and overall liking. Tasters indicated a moderate overall liking of the marinades, with no significant difference in consumer assessment of the cooked products flavour, aroma or overall liking noted (P > 0.05) (Table 8). However, the taster ratings varied, with scores from 1 to 9 given in all categories. The interquartile range reflects this, with a larger range seen for M1 in all categories. However, overall consumer opinions reflect the need for some improvement of the marinades at hand for their sensory attributes. The range of results obtained reflects those from other studies regarding the flavour of unflavoured chicken meat products, which indicate a moderate to good liking of unflavoured chicken taste or aroma. However consumer sensory assessment scores rarely exceed 8/10, or 7/9 in the case of a 9 point scale, for aroma, flavour or liking across studies utilising unaltered chicken breast meat [[55], [56], [57]].

**Table 8:** Surveyed consumer perceptions of product qualities (Mean rating on a scale of 1–9 ± St. Deviation plus interquartile Range (IQR)).

Marinade	Overall	Flavour	Aroma	Appearance	Colour
Marinade 1 *IQR*	4.1 (*± 2.2*) 2.0–6.0	4.4 (*± 2.3*) 2.0–6.0	4.8 (*± 1.8.*) 4.0–7.0	6.4 (*± 2.0*) 6.0–8.0	6.7 (*± 1.4*) 6.0–8.0
Marinade 2 *IQR*	4.7 (*± 2.4*) 2.5–6.5	4.3 (*± 2.3*) 2.0–6.0	6.0 (*± 2.1*) 5.0–8.0	5.9 (*± 1.9*) 4.5–7.0	6.2 (*± 1.9*) 5.5–7.0

Sensory perception of attribute intensity and ‘just about right’ (JAR) scores provided more reasoning for this disparity, as well as possibilities for recipe improvements. No significant difference in product acceptability was seen between marinades (P > 0.05). Many participants found that the citrus flavours such as lemon too strong for the cooked chicken, even with the reduced marination time (Table 9). This is an important consideration when designing these marinades, which might require modification of these strong flavours depending on the desired consumption of these products. This might be achieved with an agent such as olive oil, which had been incorporated into M3, but shown no antimicrobial effect.

**Table 9:** Surveyed consumer perceptions of marinade ingredient aroma and taste strength/appropriateness (Mean rating on a JAR scale of 1–5 ± St. Deviation, where 1 = Too Weak, 3 = Just Right and 5 = Too Strong). (EO = Essential oil).

Marinade	Sensory Quality	Juice Intensity	Juice Propriety	EO Intensity	EO Propriety	Herb Intensity	Herb Propriety
Marinade 1	Aroma	2.8 (*±1.3*)	3.6 (*± 1.2*)	3.3 (*± 2.3)*	2.3 (*±1.2*)	2.4 (*±1.5*)	2 (*± 1.0*)
	Taste	3.5 (*± 1.2*)	4.2 ((*± 0.8*)	3.6 (*± 2.3*)	2.5 (*± 1.1*)	2.5 (*± 1.7*)	2.1 (*± 0.9*)
Marinade 2	Aroma	2.8 (*± 1.3*)	3.7 (*± 1.0*)	3.7 (*± 2.4.*)	1.4 (*± 1.2*)	1.2 (*± 1.7*)	2 (*± 0.9*)
	Taste	3.4 (*± 1.1*)	4.1 (*± 0.9*)	4.2 (*± 2.5*)	1.4 (*± 1.1*)	1.2 (*± 1.7*)	2.1 (*± 0.9*)

The addition of salt could be considered as an additional antimicrobial hurdle and to enhance mouthfeel, sweetness, balance and saltiness of the marinades and round out overall flavour while improving flavour intensity [55]. The inclusion of sodium ions and moderate concentrations of sugar or honey (i.e. sweet flavourings) could also act to supress the perception of sourness in M1 and M2 [56]. Interestingly, assessor comments might allow for development of this idea, suggesting, for example, the incorporation of the marinated chicken into a sweetened dish or salad product to ameliorate the strong citrus flavouring. This base-level liking of the marinated meat product presents opportunities for further investigation by both food safety groups and manufacturers beyond the scope of this study, which can develop products and recipes based on these two suggested modifications.

### 3.10 Applicability of antimicrobial marinade findings

The incidence of pathogens such as *Campylobacter*, *S. enterica* and *L. monocytogenes* on poultry meat has been a point of focus for the food production industry for decades, with much effort put into reducing the frequency of contaminated products entering the market. Microbiological testing criteria have been implemented by governmental and cross-border groups such as the United States Department of Agriculture (USDA), Food Standards Australia/New Zealand (FSANZ) and European Food Safety Authority (EFSA), part of the European Union (E.U.). The E.U. has introduced guidelines limiting *Campylobacter* to 1000 CFU/g and FSANZ limiting it to 10,000 CFU/g, while all three organisations show zero tolerance for the presence of *Salmonella* or L. *monocytogenes* in fresh poultry meat [53,57,58]. However, despite the implementation of effective wash and chilling during processing, a high number of end-products can be contaminated with these pathogens (Table 10). As such, it is important to increase consumer awareness of appropriate hygiene practices in domestic food preparation settings to reduce the risk posed by products contaminated with these pathogens.

**Table 10:** Incidence of pathogen carriage on broiler products across the world.

Pathogen	Region	Proportion Contaminated Meat	Ref.
*Campylobacter* spp.	Australasia	26.2–90.00%	[[59], [60], [61]]
	Americas	18.70–59.2%	[62,63]
	Europe	17.38–80.00% 75.30%	[[64], [65], [66]]
	Ghana	38.30%	[67]
	Pakistan	29.00%	[68]
*Salmonella enterica*	Americas	19–31.50%	[63,69]
	China	26.40%	[70]
	North Africa	0.90–20.00%	[71,72]
	Europe	0.00–46.67%	[64]
	Indonesia	85.00%	[73]

Marinades were designed using the findings above, building on influences from the experiences of the researchers as well as the established MICs of the EOs of interest (Table 3). These marinades had quick effect, reducing bacterial abundance by 5–6 Log CFU/ml within 2 h of treatment. With these results in mind, chicken drumsticks inoculated with high levels of the test bacteria were marinaded and stored in chilled conditions over a ten-day period. Meat from the same processing facility which had not been inoculated with a challenge pathogen showed similar bacterial growth kinetics to the untreated control utilised in this report for TVCs, LAB and psychrophiles for the duration of the study (Results under review; Marmion et al., 2023). M1 and M2, which were based upon a mixture of citrus juices, EOs and a flavouring component, showed the ability to both significantly reduce the carriage of pathogens, and increase the shelf-life of the treated chicken from 2 days to 7 days. However, there was no significant difference in the microbial carriage of chicken treated with marinade 3. This marinade was olive oil-based, with the addition of oregano and basil oil and garlic paste, ingredients which had individually shown antibacterial effect, but did not have the same effect when mixed as a final composition.

The antibacterial application and extension of shelf-life using essential oils has been demonstrated in previous studies as part of direct use on meat products, and as part of marinades [17,18,[74], [75], [76]]. These studies tended to focus on carvacrol and thymol-containing oils such as thyme EO and oregano EO, though many of them noted the strong flavours associated with these compounds, particularly at concentrations which showed antibacterial effects. Other marinades based in thyme oil in soy sauce [75], teriyaki sauce [77], red wine [78], and a salt-thyme oil-orange oil composition [79] similarly exploited a multi-hurdle approach to successfully control microbial growth on meat, but none used the same combinations of oils, EOs and citrus juices as outlined in this report. Furthermore, the marinades employed in this study showed improved effect when compared to organic acid-only marinades employed against *C. jejuni* on fresh meat over a three day period [80].

The use of low pH through juice components may, however, be a cause for concern, and further investigations are needed to ascertain whether a risk exists for consumers. It was notable that LAB and mesophilic growth occurred in tandem in the shelf-life assay; this may be due to the acid and cold tolerance of this family of bacteria, leading to overgrowth of these microbes [81].

However, of more concern are pathogenic genera. Enteric pathogens such as *Salmonella* employ pH-dependent activation of virulence gene cassettes, such as *Salmonella* Pathogenicity Island (SPI) 1, and could lead to stress pre-conditioning which might pose a threat to consumers. There has been some increased virulence factor expression and resistance to levofloxacin and daptomycin seen in studies utilising *Listeria monocytogenes* on ceviche, which utilises similar acid stress to reduce bacterial risk. This altered resistance and virulence might affect marinade acceptability as an intervention [82]. Furthermore, acid stress has been shown to induce efflux activity in several bacterial species, which could contribute to multi-drug resistance traits in surviving cells [83,84]. However, the multi-hurdle design of these marinades, acting through several stressors on the cell, hopes to circumvent this concern. However, gene-expression and transcriptomic-level studies are required to ensure that this potential risk is not the case in surviving cells within the meat.

Given the need to develop consumer-friendly antimicrobial interventions and the potential for poor food-handling practices within the homes of consumers, interventions such as these proposed marinades offer a means to reduce the spread of foodborne pathogens which do enter the home. Food preparation, storage and cooking practices such as temperature abuse, barbecuing, insufficient cook-times, and misuse of meat thermometers can allow for the survival or proliferation of pathogens in food, which can lead to downstream illnesses in vulnerable populations [85]. Some of these practices have the potential to grow in line with projected climate change, with warm weather associated with barbecuing and subsequent foodborne illnesses in several studies [86,87]. Consumers are more accepting of natural interventions to improve food safety, which provides an opportunity for processors to add these marinades to their product ranges to mitigate consumer health concerns [88]. Many producers already incorporate marinaded products into their product lines, and the introduction or modification of recipes which enhance food safety offers a means to attract consumers who show hesitance in the face of other means of controlling food-associated disease. The added advantage of providing a wide diversification of flavours and extending the cold storage of treated meat means that the poultry could quickly effect an exciting and safe eating experience for consumers. Additionally, many noted risk-associated practices such as poor food preparation and storage could be mitigated with the introduction of similar in-home interventions to consumers. These marinades have added benefits for consumers, representing a fast-acting and low-cost intervention to improve food safety, extending the cold storage of treated meat, and acting as an easy-to-make flavouring for consumers at large. However, given the results of the preliminary taster surveys as well as the reality that this approach does not suit the needs of all consumers who may prefer unflavoured products, it is evident that while the concept provides opportunities for the maintenance of consumer safety, more work must be done to ensure the products are both safe and acceptable to the end-consumer.

## 4 Conclusions

Given the prevalence of foodborne pathogens such as *Campylobacter* and *Salmonella* in poultry products on the retail market, it is important for consumers to prepare food in a way that minimises the risk of foodborne illness. Three marinades for use on chicken meat were designed using established inhibitory concentrations of essential oils and citrus juices, as well as flavouring agents, and assessed for their effectiveness against bacterial contamination and spoilage of food. Marinades made using essential oil concentrations of 0.5% thyme oil or based upon citrus juices were found to be most effective in both reducing the prevalence of artificially inoculated *Campylobacter jejuni*, *Salmonella enterica* ser. Typhimurium and *Listeria innocua*, and increasing the shelf-life of treated meat from 2 days to 7 days. However, consumer studies indicate that more focused design steps are needed to mitigate strong flavours associated with the ingredients investigated in this paper. Once optimised, these marinades can offer a low-cost and appealing step for consumers and manufactures to improve food safety.

## Declarations

### 4.1 Author declarations

1 - Conceived and designed the experiments;

2 - Performed the experiments;

3 - Analyzed and interpreted the data;

4 - Contributed reagents, materials, analysis tools or data;

5 - Wrote the paper.

## Funding information

This project was funded by the Department of Food Agriculture and the Marine (Ireland) (Grant no. 17/F/275), under the 10.13039/100013206Food Institutional Research Measure (FIRM).

## Declaration of competing interest

The Authors declare that there are no known external or internal competing interests which may have affected the outcome of this work.
