# Supplementary material for: Green label marinades: A solution to salmonella and campylobacter in chicken products?
Source: Heliyon. 2023 Jul 4;9(7):e17655. doi: 10.1016/j.heliyon.2023.e17655 (PMC10362192; doi:10.1016/j.heliyon.2023.e17655)
Supplement: Multimedia component 1 [file mmc1.docx]

***Supplementary Table 1:*** *Active ingredient of essential oils*

| **Essential Oil** | **Plant** | **Antimicrobial Components** | **Reference** |
| --- | --- | --- | --- |
| Allspice Oil | Pimenta dioica | Eugenol | [29] |
| Anise Oil | Pimpinella anisum | α-Cymene, α-Pinene, Linalool | [30] |
| Basil Oil | Ocimum basilicum | Linalool | [31,32] |
| Betel Oil | Piper betle | Hydroxychavicol, Eugenol | [33] |
| Cinnamon Oil | Cinnamomum zeylanicum | Cinnamaldehyde | [31] |
| Cumin Oil | Cuminum cyminum | γ-Terpinene, α- and β-Pinene, Cuminic aldehyde, Linalool | [25] |
| Eucalyptus Oil | Eucalyptus globulus | 1,8-cineole, Citronellal, Citronellol, Citronellyl acetate, p-Cymene, Eucamalol, Limonene, Linalool, β- Pinene, γ-terpinene | [34] |
| Fennel Oil | Foeniculum vulgare | Limonene, α-and β-Pinene, γ-Terpinene | [30] |
| Garlic Oil | Allium sativum | Allcilin | [18] |
| Lavender Oil | Lavandula officinalis | Linalool, Linalyl acetate | [35] |
| Lemongrass oil | *Cymbopogon citratus* | Citral, Geraniol, Neral | [36] |
| Marjoram Oil | Origanum majorana | Carvacrol, Thymol, 1,8-Cineole, Limonene, α- and β-Pinene, Linalool | [30] |
| Oregano oil | Origanum vulgarae | Carvacrol, Thymol | [18] |
| Peppermint Oil | Mentha piperita | Menthol, Menthone, Methyl acetate, Limonene | [37] |
| Rosemary Oil | Salvia rosemarinus | 1,8-Cineole, Camphor, α- and β-Pinene | [29] |
| Sage Oil | Salvia officinalis | α- and β-Pinene, Camphene, α-Terpineol, Linalool | [38] |
| Thyme Oil | Thymus vulgaris | Carvacrol, Thymol | [18] |
| Winter Savoury Oil | Satureja montana | Thymol, Carvacrol | [39] |
