# Supplementary material for: Green label marinades: A solution to salmonella and campylobacter in chicken products?
Source: Heliyon. 2023 Jul 4;9(7):e17655. doi: 10.1016/j.heliyon.2023.e17655 (PMC10362192; doi:10.1016/j.heliyon.2023.e17655)
Supplement: Multimedia component 2 [file mmc2.docx]

***Supplementary Table 2.*** *Bacterial strain type and growth conditions*

| **Species** | **Seq. type** | **Temp.** | **Medium** | **Oxygen** |
| --- | --- | --- | --- | --- |
| *Bacillus subtilis* | DSM10 | 37 ℃ | Tryptic Soya Broth | (+) |
| *Brochothrix thermosphacta* | DSM 0171 | 30 ℃ | Tryptic Soya Broth | (+) |
| *Campylobacter jejuni* | NCTC 11168 | 42 ℃ | Bolton Broth | (-) |
| *Escherichia coli* | ATCC 8739 | 37 ℃ | Tryptic Soya Broth | (+) |
| *Listeria monocytogenes* | LO28 | 30 ℃ | Tryptic Soya Broth | (+) |
| *Listeria innocua* | DSM 20649 | 37 ℃ | Tryptic Soya Broth | (+) |
| *Pseudomonas aeruginosa* | ATCC 27853 | 37 ℃ | Tryptic Soya Broth | (+) |
| *Pseudomonas fluorescens* | Food Isolate | 37 ℃ | Tryptic Soya Broth | (+) |
| *Salmonella enterica* ser. Dublin | Isolate | 37 ℃ | Tryptic Soya Broth | (+) |
| *Salmonella enterica* ser. Enterica | DSM 19587 | 37 ℃ | Tryptic Soya Broth | (+) |
| *Salmonella enterica* ser. Typhimurium | ATCC 14028 | 37 ℃ | Tryptic Soya Broth | (+) |
