# Supplementary material for: Green label marinades: A solution to salmonella and campylobacter in chicken products?
Source: Heliyon. 2023 Jul 4;9(7):e17655. doi: 10.1016/j.heliyon.2023.e17655 (PMC10362192; doi:10.1016/j.heliyon.2023.e17655)
Supplement: Multimedia component 3 [file mmc3.docx]

***Supplementary Table 3.*** *Summary of media used to enumerate and or enrich for bacterial pathogens and spoilage bacteria in chicken meat samples.*

| **Medium** | **Supplement** | **Target** | **Temperature** | **Duration** |
| --- | --- | --- | --- | --- |
| ***Enumeration Media*** |  |  |  |  |
| Plate Count Agar (PCA) | None | Mesophiles (TVCs) | 37 °C | 48 Hrs |
|  |  | Psychrophiles | 4 °C | 240 Hrs |
| DeMann, Rogosa and Sharpe Agar (MRS) | None | Lactic Acid Bacteria | 30 °C | 72 Hrs |
| Ottaviani-Agosti Agar | Ottaviani-Agosti Supplement | *Listeria innocua* | 37 °C | 24-48 Hrs |
| Modified Charcoal Cefoperazone Deoxycholate Agar (mCCDA) | mCCDA Supplement | *Campylobacter* spp. * | 37 °C | 48 Hrs |
| Xylose Lysine Deoxycholate Agar (XLD) | None | *Salmonella* spp. | 37 °C | 24 Hrs |
| ***Enrichment Media*** |  |  |  |  |
| Bolton Broth | Bolton supplement  Lysed horse blood | *Campylobacter* spp. | 37 °C | 24 (Hrs) |
| Rappoport-Vassiliadis Broth | None | *Salmonella* spp. | 37 °C | 24 (Hrs) |
| Tryptone Soya Broth | None | *Listeria innocua* | 30 °C | 24 (Hrs) |

**Campylobacter* spp. growth requires storage under microaerobic conditions; provided with Campygen™ sachets (Thermo-Fisher Scientific Ltd., Basingstoke, U.K.).
