# Supplementary material for: Green label marinades: A solution to salmonella and campylobacter in chicken products?
Source: Heliyon. 2023 Jul 4;9(7):e17655. doi: 10.1016/j.heliyon.2023.e17655 (PMC10362192; doi:10.1016/j.heliyon.2023.e17655)
Supplement: Multimedia component 4 [file mmc4.docx]

***Supplementary Table 4.*** *Thickening agents and effective concentrations investigated for incorporation into the final marinade compositions.*

| **Thickening agent** | **Concentrations (% w/v)** | **Mixing Temp.** | **pH Adjustment?** |
| --- | --- | --- | --- |
| Arabic Gum | 2.0, 5.0 | 60, 70 °C | No |
| Carrageenan | 0.5, 1.0, 1.5 | 80 °C | Yes |
| Gelatin | 1.0, 1.5 % | 60, 80 °C | Yes |
| Guar Gum | 0.5, 1.0 % | 25, 37 °C | No |
