# Supplementary material for: Green label marinades: A solution to salmonella and campylobacter in chicken products?
Source: Heliyon. 2023 Jul 4;9(7):e17655. doi: 10.1016/j.heliyon.2023.e17655 (PMC10362192; doi:10.1016/j.heliyon.2023.e17655)
Supplement: Multimedia component 5 [file mmc5.docx]

***Supplementary Table 5.*** *Antimicrobial activity of final marinade compositions against hospital and broiler-sourced Campylobacter jejuni isolates. (+) indicates effect. (–) indicates no effect.*

| **Isolate** | **Source** | **M1** | **M2** | **M3** |
| --- | --- | --- | --- | --- |
| 1 | Broiler | + + + | + + + | - - - |
| 2 | Broiler | + + + | + + + | - - - |
| 3 | Clinical | + + + | + + + | - - - |
| 4 | Clinical | + + + | + + + | + - - |
| 5 | Clinical | + + + | + + + | - - - |
| 6 | Clinical | + + + | + + + | - - - |
| 7 | Broiler | + + + | + + + | - - - |
| 8 | Broiler | + + + | + + + | - - - |
| NCTC11168 | Known Strain | + + + | + + + | - - - |
