# Supplementary material for: Green label marinades: A solution to salmonella and campylobacter in chicken products?
Source: Heliyon. 2023 Jul 4;9(7):e17655. doi: 10.1016/j.heliyon.2023.e17655 (PMC10362192; doi:10.1016/j.heliyon.2023.e17655)
Supplement: Multimedia component 6 [file mmc6.docx]

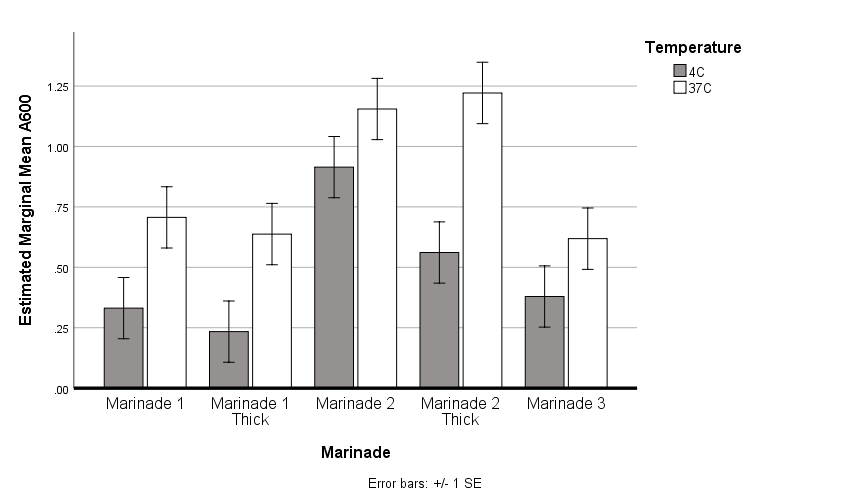


(A)


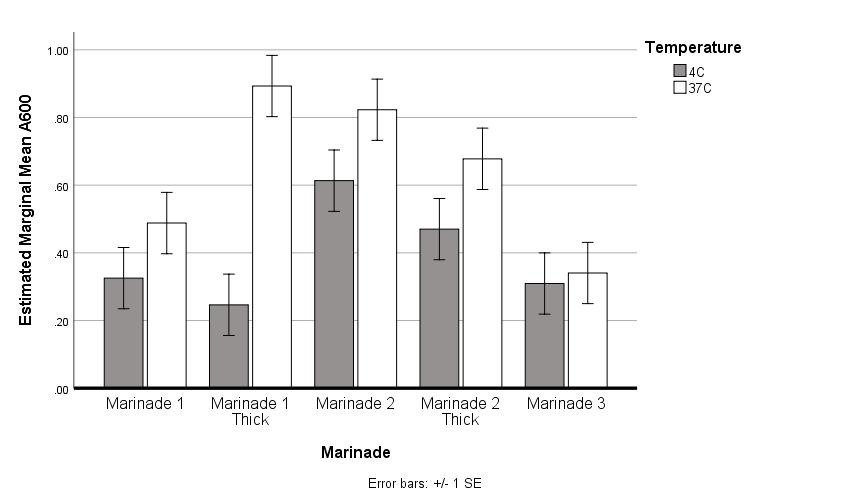


(B)


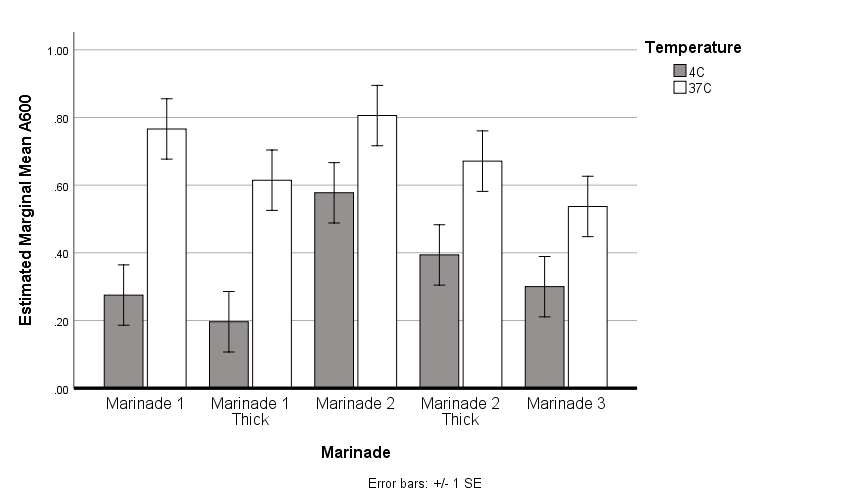


(C)


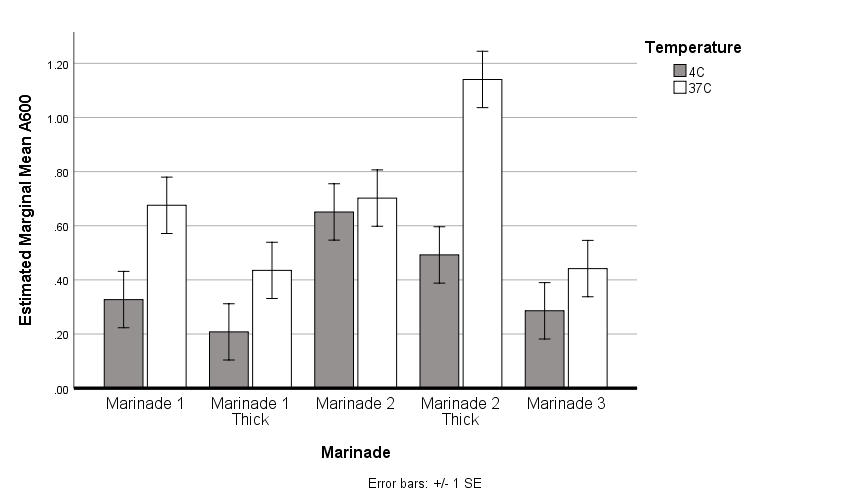


(D)

***Supplementary Figure 1:*** *Biofilm formation by (A) Salmonella enterica (B) Listeria innocua (C) Escherichia coli (D) Bacillus subtilis in unthickened and thickened marinade compositions at 4 ℃ and 37 ℃.*
